# Modulation of 14-3-3/Phosphotarget Interaction by Physiological Concentrations of Phosphate and Glycerophosphates

**DOI:** 10.1371/journal.pone.0072597

**Published:** 2013-08-19

**Authors:** Nikolai N. Sluchanko, Natalia A. Chebotareva, Nikolai B. Gusev

**Affiliations:** 1 Laboratory of Molecular Organization of Biological Structures, A.N. Bach Institute of Biochemistry of the Russian Academy of Sciences, Moscow, Russian Federation; 2 Department of Biochemistry, School of Biology, Moscow State University, Moscow, Russian Federation; Case Western Reserve University, United States of America

## Abstract

Molecular mechanisms governing selective binding of a huge number of various phosphorylated protein partners to 14-3-3 remain obscure. Phosphate can bind to 14-3-3 and therefore being present at high intracellular concentration, which undergoes significant changes under physiological conditions, phosphate can theoretically regulate interaction of 14-3-3 with phosphorylated targets. In order to check this hypothesis we analyzed effect of phosphate and other natural abundant anions on interaction of 14-3-3 with phosphorylated human small heat shock protein HspB6 (Hsp20) participating in regulation of different intracellular processes. Inorganic phosphate, glycerol-1-phosphate and glycerol-2-phosphate at physiologically relevant concentrations (5-15 mM) significantly destabilized complexes formed by 14-3-3ζ and phosphorylated HspB6 (pHspB6), presumably, via direct interaction with the substrate-binding site of 14-3-3. Phosphate also destabilized complexes between pHspB6 and 14-3-3γ or the monomeric mutant form of 14-3-3ζ. Inorganic sulfate and pyrophosphate were less effective in modulation of 14-3-3 interaction with its target protein. The inhibitory effect of all anions on pHspB6/14-3-3 interaction was concentration-dependent. It is hypothesized that physiological changes in phosphate anions concentration can modulate affinity and specificity of interaction of 14-3-3 with its multiple targets and therefore the actual phosphointeractome of 14-3-3.

## Introduction

Members of eukaryotic 14-3-3 protein family are well-known universal proteins having an expanding repertory of functions from regulation of apoptosis or cell division to chaperone-like activity. Universality in 14-3-3 action might be associated with their ability to interact with a huge number of different protein partners involved in multiple cellular processes [1]. In the latest studies the number of predicted 14-3-3 partners reaches ~700 [2]. 14-3-3, usually presented as several isoforms [3,4], predominantly interacts with proteins phosphorylated at serine or threonine in certain consensus sequences [5,6]. In these sequences phosphoserine (or phosphothreonine) is preceded by positively charged Arg/Lys in position -3 or -4 and has Pro/Gly in position +2 (the so-called motifs I and II) [6,7]. Alternatively, phosphorylated serine (or threonine) is located at the very C-terminal end of a protein partner (motif III) [5].

Crystallographic studies revealed that 14-3-3 monomers tend to form dimers stabilized by interaction of their N-termini [8]. Each 14-3-3 monomer contains substrate-binding site responsible for binding of phosphorylated peptide of a client protein [8]. This multisite interaction is amphipathic in nature [8]. However, electrostatic interactions of phosphate moiety with positively charged triad of Lys49, Arg56, Arg127 and Tyr128 [7] seem to play crucial role since non-phosphorylated peptides of the same composition fail as a rule to bind to 14-3-3. Therefore, phosphorylation of client proteins is especially important for regulation of their interaction with 14-3-3.

The intracellular concentration of 14-3-3 in certain tissues is rather high [9], however, is much smaller than the total concentration of hundreds of 14-3-3 potential protein targets [10,11]. Therefore, an intriguing question arises, what determines selectivity and specificity of 14-3-3. Obviously, the primary structure of phosphorylated sites of target proteins, their compartmentalization and concentration (availability) might regulate the specificity of interaction of 14-3-3 with their diverse targets. However, we suppose that this is insufficient and there should be other general mechanisms providing for selectivity and specificity of 14-3-3 interaction with its multiple potential partners and eliminating binding of undesirable targets usually having low affinity.

Along with phosphorylated proteins there are a lot of organic and inorganic phosphate-containing compounds presented in the cell. Phosphate is considered as the most abundant cellular anion [12]. The concentration of inorganic phosphate (P_i_) is in the range of mM (or tens of mM) [13] and undergoes significant changes under physiological conditions [13,14]. For example, intracellular levels of inorganic phosphate in fatiguing muscle can reach 24-28 mM [15]. In addition, intracellular concentration of other phosphate-containing substances (e.g., phosphocreatine, nucleotides, phosphorylated carbohydrates, glycerophosphates, phospholipids, etc.) is also in the range of mM or tens of mM [16].

Recently, we found that P_i_ increases stability of 14-3-3ζ monomers and may inhibit its interaction with partner proteins probably competing with protein substrate [17]. Sparse data of the literature indicated that P_i_ affects interaction of 14-3-3 with certain phosphorylated targets [18,19]. However, these observations were not thoroughly analyzed and the specificity of phosphate was neither established nor explained.

Therefore, in this study we analyzed the effect of phosphate and certain other anions containing isosteric group on the interaction of 14-3-3 with phosphorylated small heat shock protein HspB6 (Hsp20) whose interaction with 14-3-3 is believed to be important for regulation of different cellular processes [20]. The data obtained by a combination of hydrodynamic techniques indicate that phosphate and glycerophosphates in physiologically relevant concentrations (5-15 mM) can in concentration-dependent manner modulate interaction of 14-3-3 with its phosphorylated protein partner. We suppose that physiological changes of phosphate anions concentration may be considered as a dynamic ‘filter’ affecting binding of 14-3-3 to different target proteins and therefore the actual 14-3-3 phosphointeractome.

## Materials and Methods

### Materials

All chemicals (phosphate (P_i_), pyrophosphate (PP_i_), sulfate, glycerol-1-phosphate (Gl-1-P), glycerol-2-phosphate (Gl-2-P), sodium chloride, Tris, EDTA) were of the highest purity and quality available. All solutions in the study were prepared on the milliQ-quality water (18.3 MΩ/cm) and filtered through the 0.22 µm Millipore filter system before use.

### Proteins

Cloning, expression and purification of the full-length untagged human wild type 14-3-3γ (Uniprot ID P61981), wild type 14-3-3ζ (Uniprot ID P63104), its mutant form which is unable to form dimers, 14-3-3ζ_m_ [21], and wild type small heat shock protein HspB6 (Hsp20) (Uniprot ID O14558) was performed as described earlier [17,22,23]. The recombinant active catalytic subunit of mouse cAMP-dependent protein kinase (PKA) was obtained as described in [24]. Protein concentration was determined spectrophotometrically and is indicated in μM per monomer. All proteins were electrophoretically homogeneous.

### HspB6 phosphorylation by PKA

HspB6 (59 µM) was phosphorylated by PKA in 50 mM Tris-HCl buffer, pH 7.4, containing 150 µM ATP, 4 mM MgCl_2_, 10 mM NaCl and 2 mM dithiothreitol (DTT) for 1 h at 37^o^C. Reaction was stopped by addition of 10 mM EDTA and freezing at -20^o^C. Under conditions used the stoichiometry of phosphorylation was close to 1 mole P_i_/mole of HspB6 [22,23]. The presence of covalently bound phosphate was confirmed by tandem mass spectrometry on MALDI ultra-fleXtreme mass-spectrometer (Bruker).

### Analytical size-exclusion chromatography (SEC)

SEC was applied for investigation of the effect of different anions (phosphate, sulfate, pyrophosphate, glycerol-1-phosphate and glycerol-2-phosphate) on the interaction between 14-3-3ζ, its monomeric form, 14-3-3ζ_m_, or 14-3-3γ and phosphorylated HspB6 (pB6) [17,21–23]. To investigate the effect of particular anion we performed series of experiments at different anion concentrations (0-75 mM) preparing series of dilutions of a starting 75 mM anion solution containing also 0.1 mM EDTA and 15 mM β-mercaptoethanol (ME). Dilutions were made with 35 mM Tris-HCl buffer, pH 7.5, containing 0.1 mM EDTA and 15 mM ME. After dilution pH of solution was adjusted to 7.5 and NaCl was added so that ionic strength of all buffers was identical. Each series included chromatographic analysis of isolated 14-3-3 species, isolated pHspB6 or the mixture of pHspB6 and different species of 14-3-3. Concentrations of proteins are indicated in figure legends. Protein samples (50 µl) were incubated for 30 min at 37^o^C in the appropriate column buffer and loaded on a ProSEC300S column (Varian) preceded by a ProSEC300S Guard column (Varian) pre-equilibrated with the same buffer. SEC was performed on a HPLC chromatographic system equipped with a ProStar 325 UV–Vis detector (Varian). All experiments were performed at an ambient temperature at a flow rate of 1 ml/min. If necessary, fractions (130 µl) were collected and analyzed by SDS gel-electrophoresis.

### Analytical ultracentrifugation

Sedimentation velocity experiments were carried out at 20^o^C in a Model E analytical ultracentrifuge (Beckman), equipped with absorbance optics, a photoelectric scanner, a monochromator and a computer online. A four-hole rotor An-F Ti and 12 mm double sector cells were used. The sedimentation profiles of the mixtures of 14-3-3ζ (24 µM or 48 µM) and pHspB6 (22 µM) in 35 mM Tris-HCl buffer, pH 7.5, containing 100 mM NaCl, 0.1 mM EDTA and 15 mM ME (buffer 1) or in the same buffer containing 37.5 mM of inorganic phosphate (buffer 2, pH 7.5) or 37.5 mM of glycerol-2-phosphate (buffer 3, pH 7.5) instead of NaCl were recorded by measuring the absorbance at 290 nm against appropriate buffer (buffer 1, 2 or 3) containing all aforementioned additives except of the proteins. All cells were scanned simultaneously with 3 min interval. The sedimentation coefficients were estimated from the differential sedimentation coefficient distribution [*c*(*s*) versus *s*] using SEDFIT program [25] and the *c*(s) distributions were transformed into *c*(M) distributions. The sedimentation coefficients were corrected to the standard conditions using SEDFIT and SEDNTERP [26] programs.

## Results

**Figure 1:**
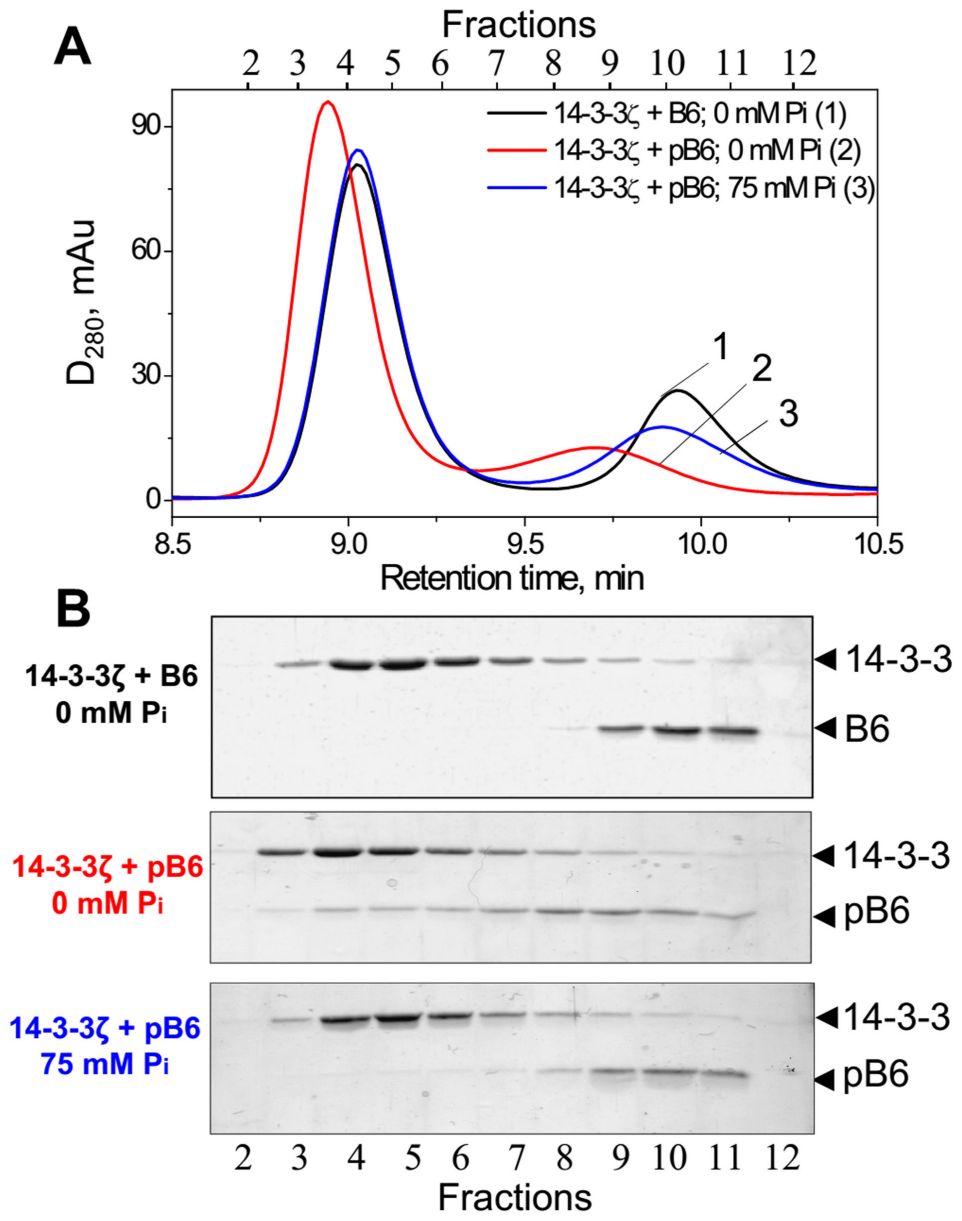
Analytical SEC of 14-3-3ζ, unphosphorylated HspB6 (B6) or phosphorylated HspB6 (pB6) and their mixtures in the presence or in the absence of phosphate (P_i_). (**A**) Elution profiles of the mixture of 14-3-3ζ and either of unphosphorylated (B6, curve 1) or phosphorylated (pB6, curve 2) HspB6 (28 µM of each) performed in Tris-buffer or of the mixture of 14-3-3ζ and pHspB6 performed in phosphate-containing buffer (curve 3). Representative results of four independent experiments are presented. Protein composition of the fractions was analyzed by SDS gel-electrophoresis (**B**). Positions of 14-3-3 (~30 kDa) and HspB6 (~20 kDa) are indicated by arrows.

As reported earlier, unphosphorylated HspB6 (B6) does not interact with 14-3-3 [17,21–23]. Therefore if the mixture of 14-3-3ζ and HspB6 proteins was loaded on the column we detected two separate peaks containing either 14-3-3ζ or HspB6 (Figure 1A; curve 1 and 1B). Phosphorylated HspB6 (pHspB6) interacts with 14-3-3ζ and therefore the amplitude of the peak of pHspB6 was decreased and a new peak with smaller retention time was detected on the elution profile (Figure 1A; curve 2). As expected this peak contained both proteins (Figure 1B). However, when the same mixture of pHspB6 and 14-3-3ζ was subjected to SEC in the presence of P_i_, the elution profile was very similar to that of non-interacting 14-3-3ζ and B6 and almost all pHspB6 returned to its original position on the chromatogram (Figure 1B, fractions 9-11).

**Figure 2:**
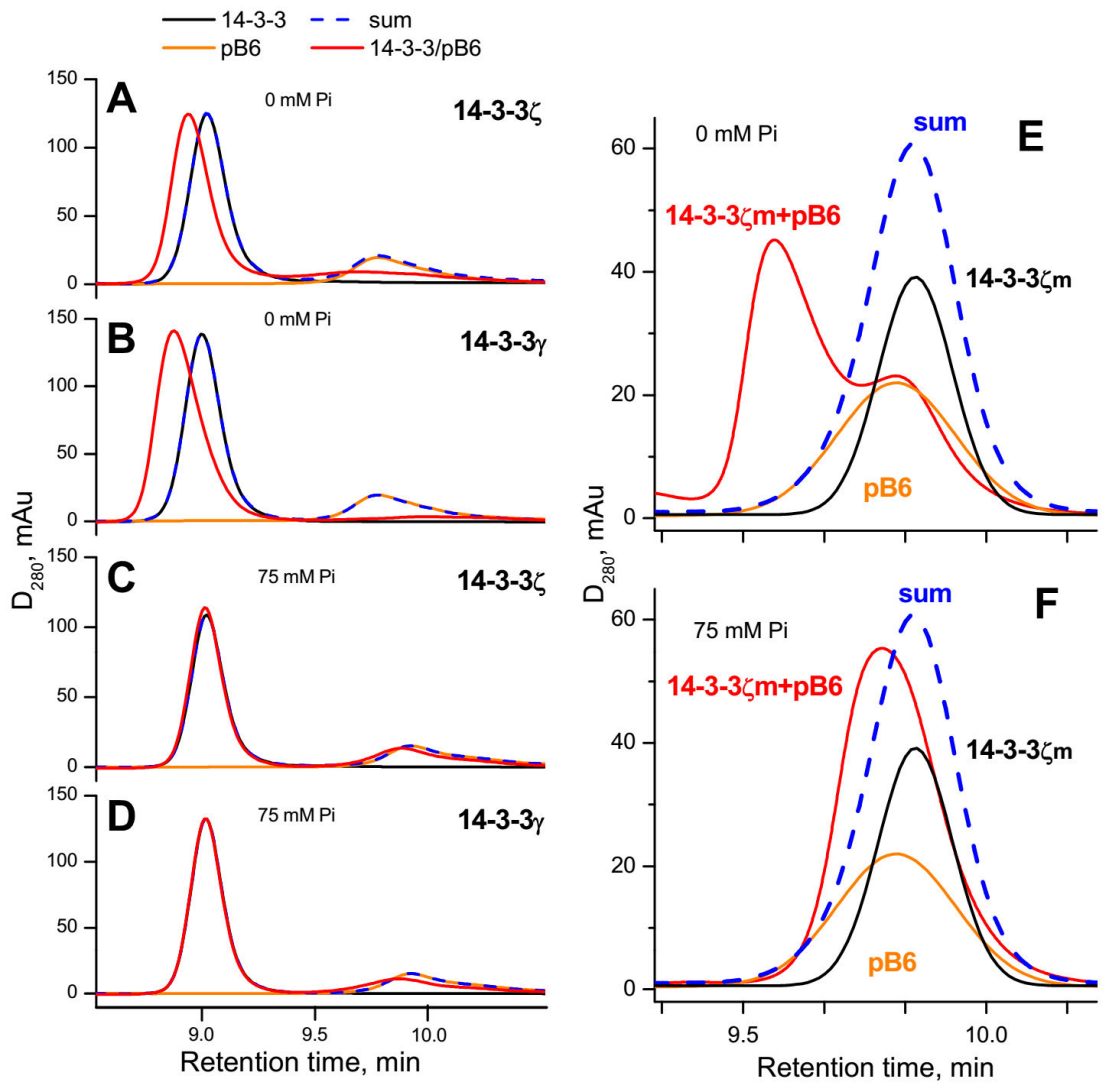
Effect of phosphate (P_i_) on the interaction of pHspB6 with the wild type 14-3-3ζ, 14-3-3γ or with the 14-3-3ζ mutant being unable to form dimers (14-3-3ζ_m_) studied by SEC. 14-3-3ζ (39 µM) (**A**, **C**), 14-3-3γ (39 µM) (**B**, **D**), 14-3-3ζ_m_ (13 µM) (**E**, **F**) alone (black lines) or pHspB6 (21 µM) alone (orange lines) or the mixtures of pHspB6 and different 14-3-3 proteins (red lines) were pre-incubated and subjected to SEC in the absence (**A**, **B**, **E**) or in the presence (**C**, **D**, **F**) of 75 mM phosphate in elution buffer. Blue dashed lines represent algebraic sum of elution profiles of isolated 14-3-3 and isolated pHspB6 and indicate lack of 14-3-3/pB6 interaction. Representative results of three independent experiments are presented.

In a good agreement with our earlier published data [23], 14-3-3γ also formed tight complexes with pHspB6 in Tris-buffer (Figure 2). However, these complexes almost completely dissociated to their components if SEC was performed in the presence of 75 mM phosphate (Figure 2). In this respect both 14-3-3γ and 14-3-3ζ demonstrated similar behavior (Figure 2A–D). Continuing our investigation we analyzed interaction of pHspB6 with 14-3-3ζ mutant which is unable to form stable dimers (the so-called monomeric mutant 14-3-3ζ_m_). This monomeric form of 14-3-3 is functionally active and specifically interacts only with phosphorylated form of HspB6 [17,21]. This interaction results in formation of the complex which is eluted as a well-separated peak with small retention time on the elution profile (Figure 2E). This peak almost completely disappeared if SEC was performed in the presence of 75 mM of phosphate (Figure 2F) thus indicating that inorganic phosphate significantly destabilizes complexes formed by 14-3-3ζ_m_ and pHspB6. Qualitatively similar results were obtained when glycerol-2-phosphate was used instead of phosphate (not shown).

Since all conditions of our SEC experiments (temperature, ionic strength and pH) were kept constant, we concluded that inorganic phosphate itself destabilizes the 14-3-3/phosphotarget complexes and induces its dissociation, presumably, via direct interaction with the substrate-binding site of 14-3-3. In order to check this conclusion we analyzed the interaction of 14-3-3ζ and pHspB6 in series of SEC experiments with different anions varying their concentration in elution buffer.

**Figure 3:**
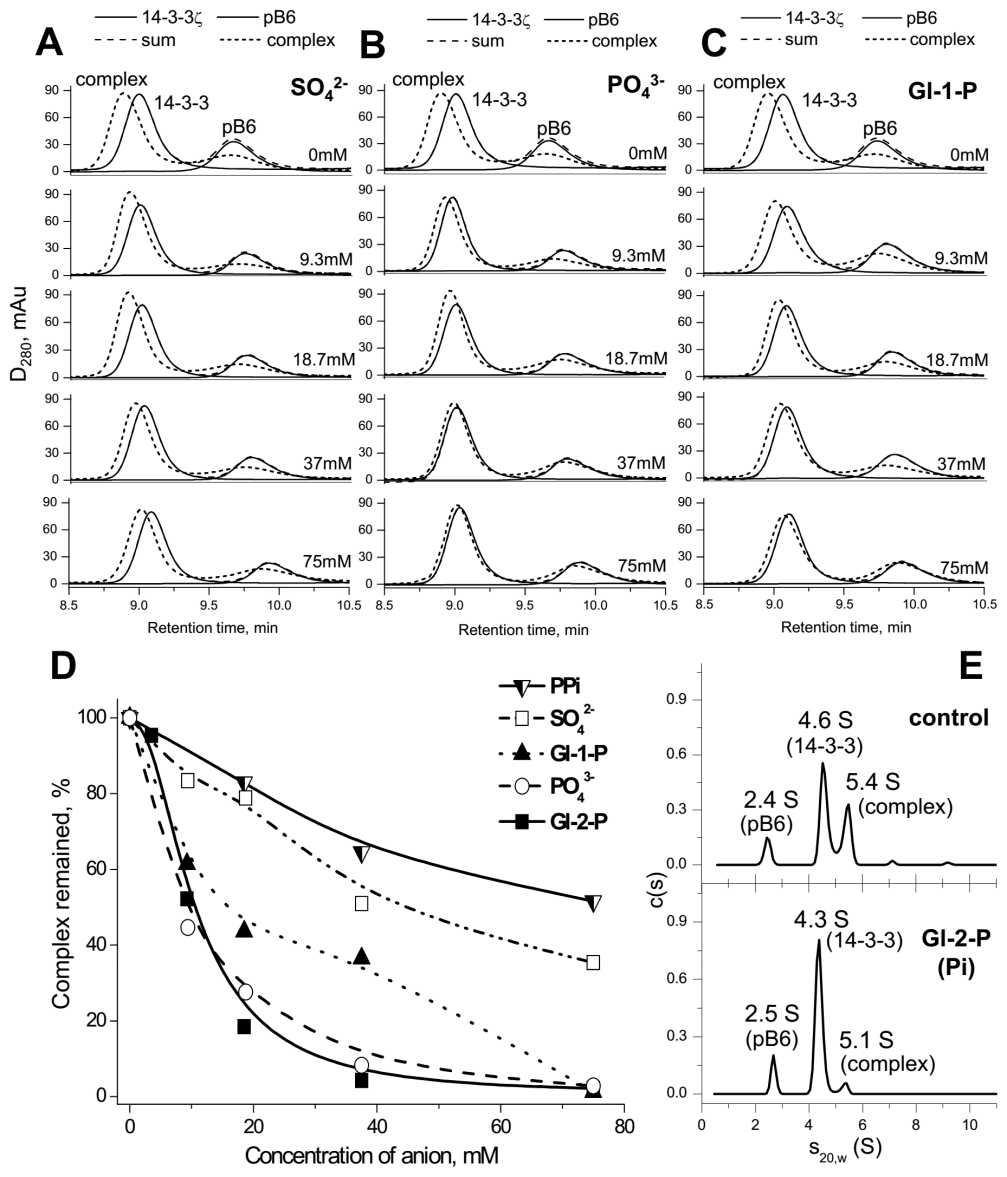
Modulation of 14-3-3ζ/phosphotarget interaction by different anions. **A**–**C**, Effect of different concentrations of sulfate (**A**), phosphate (**B**) or glycerol-1-phosphate (**C**) on the 14-3-3ζ/pHspB6 interaction studied by SEC. Typical elution profiles of 14-3-3ζ, pHspB6 (solid lines: 14-3-3 and pB6), or the mixture of interacting proteins (dotted lines) are superimposed. The algebraic sum of elution profiles of isolated proteins indicating lack of 14-3-3/pB6 interaction (dashed lines) and concentration of anions in the column buffer are also indicated. (**D**) Estimation of the inhibitory action of different anions studied by SEC. The two main parameters of 14-3-3ζ/pHspB6 interaction detected by SEC, i.e. the shift of the peak of the complex relative to the 14-3-3 position (P1) and the decrease of the amplitude of the peak of HspB6 reflecting its migration towards the position of the complex (P2) were calculated for each concentration of the corresponding anion. The product P1*P2 reflected the efficiency of complex formation and was used for estimation of destabilizing effect of different anions on the 14-3-3ζ/pHspB6 interaction. The product of maximal shift (P1) and maximal decrease of the HspB6 peak (P2) observed in the absence of anions was taken as 100%. Representative data of two independent experiments performed at each anion concentration are presented and deviations of calculated P1*P2 values were less than 10%. (**E**) The *c*(s) distributions for the mixture of 14-3-3ζ (24 µM) and pHspB6 (22 µM) in the absence (control) or in the presence of 37.5 mM glycerol-2-phosphate (Gl-2-P) (lower panel). The effect of 37.5 mM P_i_ was similar and is not shown.

Sulfate up to 75 mM only slightly affected the 14-3-3ζ/pHspB6 interaction (Figure 3A). At the same time even much smaller concentrations of P_i_ (e.g., 9.3 and 18.7 mM) induced pronounced changes of the elution profiles indicating dissociation of the 14-3-3ζ/pHspB6 complex (Figure 3B). The difference in the effects of these isosteric anions might be explained by the differences in their sizes resulting in better fitting of phosphate than sulfate into the substrate-binding site of 14-3-3. Similar results were obtained in the case of glycerol-1-phosphate, which even at 9-18 mM concentration induced significant destabilization of the 14-3-3ζ/pHspB6 complex (Figure 3C). The effect of glycerol-2-phosphate commonly used as a source of phosphate group, was substantially the same as of inorganic phosphate or glycerol-1-phosphate (not shown). At the same time even at rather high concentrations pyrophosphate (PP_i_) was ineffective in modulation of the 14-3-3ζ/pHspB6 interaction (not shown). This is probably due to the fact that phosphoether bonds of glycerophosphates are identical to those of phosphorylated proteins and are different from anhydrous bonds presented in pyrophosphate. Therefore these compounds can differently fit to the substrate-binding site of 14-3-3.

In order to estimate and compare efficiency of different anions in modulation of the 14-3-3ζ/pHspB6 interaction we measured the differences in retention times for the 14-3-3ζ/pHspB6 complex and 14-3-3ζ at given anion concentration (parameter P1). In addition, we measured the decrease of the amplitude of the peak of pHspB6 in the presence of different concentrations of analyzed anions (parameter P2). The product of these two parameters (P1*P2) reflecting formation of the 14-3-3ζ/pHspB6 complex was used to estimate the influence of analyzed anions on the 14-3-3ζ/pB6 interaction. The data of Figure 3D indicate that P_i_ and glycerophosphates even at concentration of 5-10 mM significantly destabilize 14-3-3ζ/pHspB6 complexes, whereas pyrophosphate and sulfate at much higher concentrations were less effective (Figure 3B–D).

Analytical ultracentrifugation was used as an independent method in order to confirm the data of SEC 3 peaks were detected on the *c*(*s*) distribution for the mixture of pHspB6 and 14-3-3ζ in Tris-buffer (Figure 3E; control). Estimation of the molecular masses of species using *c*(M) distributions indicated that the 2.4-2.5 S peak (31 ± 2 kDa) corresponds to dimeric pB6, whereas two other peaks with 4.3-4.6 S (70 ± 4 kDa) and 5.1-5.4 S (91 ± 4 kDa) most likely correspond to dimeric 14-3-3ζ and the 14-3-3ζ/pHspB6 complexes, respectively. Such assignment of the peaks was verified in the experiment performed at twice as high concentration of 14-3-3ζ and the same concentration of pHspB6 (not shown).

Sedimentation velocity analysis of the mixture of 14-3-3ζ and pHspB6 in the presence of P_i_ or glycerol-2-phosphate revealed dramatic decrease of the 5.1-5.4 S peak (Figure 3E; low panel), thus indicating that anions under investigation destabilize 14-3-3ζ/pHspB6 complexes and induce its dissociation.

The data presented indicate that phosphate-containing compounds in mM concentrations strongly affect the interaction of 14-3-3ζ with phosphorylated HspB6 inducing dissociation of pHspB6/14-3-3 complexes in a concentration-dependent manner.

We suppose that the effect of phosphate is general and can be observed in the case of 14-3-3 interaction with different phosphorylated protein partners. For instance, our preliminary data indicate that inorganic phosphate in millimolar concentration also destabilizes complexes formed by 14-3-3ζ and phosphorylated human tau protein (not shown). The data of literature also indicate that phosphate attenuates interaction of 14-3-3 with nitrate reductase and arylalkylamine N-acetyltransferase [18,19].

We suppose that the efficiency of phosphate-dependent inhibition of 14-3-3/target interaction depends on the affinity of 14-3-3 to a particular target. In this case increase of phosphate concentration observed under certain physiological conditions (e.g., after muscle contraction [13]) will selectively decrease (or completely exclude) interaction of 14-3-3 with protein targets having low affinity to 14-3-3 or presented at low concentration. In any case phosphate-containing buffers should be used with caution in any investigations dealing with analysis of interaction of 14-3-3 with different targets.

## Discussion

The multifunctional family of 14-3-3 interacts with more than 700 predominantly phosphorylated protein targets [2], and the borders of 14-3-3 interactome expands all the time. Although in some organs intracellular 14-3-3 concentration is rather high [9], the total concentration of potential protein targets significantly exceeds that of 14-3-3. Therefore the cell must have evolved a special mechanism regulating efficiency and specificity of 14-3-3/target interaction.

The data of literature indicate that crystals of 14-3-3 can contain sulfate or phosphate ions [7,27] which may bind to 14-3-3 [28]. It was postulated that the primary binding of target proteins to 14-3-3 derives from electrostatic interactions [29]. Therefore 14-3-3/target interaction depends on ionic strength and should be especially sensitive to isosteric anions which can compete with phosphorylated sites of target proteins.

Phosphate is one of the most abundant anions in the living cell. Concentration of P_i_ in different muscle is in the range of 2-8 mM, that of sugar-phosphates is about 3-5 mM, and ATP and phosphocreatine – in the range of 3-7 and 20-30 mM, respectively [13,15]. Moreover, in the course of prolonged muscle contraction the concentration of P_i_ rises up to 20-25 mM and the concentration of sugar-phosphate – up to 10-12 mM [13,15]. P_i_ and phosphocreatine levels can also undergo several-fold changes during ischemia [14].

In this study we show that inorganic phosphate induces dissociation of complexes formed by phosphorylated HspB6 and 14-3-3γ, 14-3-3ζ or its monomeric mutant 14-3-3ζ_m_ (Figure 2). Moreover, we find that physiologically relevant concentrations of phosphate and glycerophosphates inhibit the 14-3-3ζ/pHspB6 interaction in concentration-dependent manner (Figure 3). These findings and the data of literature provide solid basis for a hypothesis that phosphate can modulate 14-3-3/targets interaction. We suppose that competing with protein targets, phosphate-containing compounds and inorganic phosphate can decrease or even prevent interaction of 14-3-3 with those protein targets that have low affinity and/or are presented at low concentration. This can significantly improve selectivity and specificity of 14-3-3 and decrease the number of protein targets being able to form stable complexes with 14-3-3 affecting its phosphointeractome upon physiological changes of phosphate levels.

Not only P_i_ but other phosphate-containing molecules can serve as regulators of 14-3-3. Besides the effect of phosphate and glycerophosphates shown in this study, it was reported very recently that phosphatidic acid can also regulate interaction and regulation of plant H^+^-ATPase by 14-3-3 [30].

Further investigations are needed in order to elucidate molecular mechanisms underlying the effect of various phosphate-containing compounds on interaction of 14-3-3 with different target proteins.

## References

[1] Mackintosh C (2004) Dynamic interactions between 14-3-3 proteins and phosphoproteins regulate diverse cellular processes. Biochem J 381: 329-342. doi:10.1042/BJ20031332. PubMed: 15167810. 15167810 10.1042/BJ20031332 PMC1133837

[2] Uhart M, Bustos DM (2013) Human 14-3-3 paralogs differences uncovered by cross-talk of phosphorylation and lysine acetylation. PLOS ONE 8: e55703. doi:10.1371/journal.pone.0055703. PubMed: 23418452. 23418452 10.1371/journal.pone.0055703 PMC3572099

[3] Aitken A (2006) 14-3-3 proteins: a historic overview. Semin Cancer Biol 16: 162-172. doi:10.1016/j.semcancer.2006.03.005. PubMed: 16678438. 16678438 10.1016/j.semcancer.2006.03.005

[4] Sluchanko NN, Gusev NB (2010) 14-3-3 proteins and regulation of cytoskeleton. Biochemistry (Mosc) 75: 1528-1546. doi:10.1134/S0006297910130031. PubMed: 21417993. 21417993 10.1134/s0006297910130031

[5] Ganguly S, Weller JL, Ho A, Chemineau P, Malpaux B et al. (2005) Melatonin synthesis: 14-3-3-dependent activation and inhibition of arylalkylamine N-acetyltransferase mediated by phosphoserine-205. Proc Natl Acad Sci U S A 102: 1222-1227. doi:10.1073/pnas.0406871102. PubMed: 15644438. 15644438 10.1073/pnas.0406871102 PMC544185

[6] Rittinger K, Budman J, Xu J, Volinia S, Cantley LC et al. (1999) Structural analysis of 14-3-3 phosphopeptide complexes identifies a dual role for the nuclear export signal of 14-3-3 in ligand binding. Mol Cell 4: 153-166. doi:10.1016/S1097-2765(00)80363-9. PubMed: 10488331. 10488331 10.1016/s1097-2765(00)80363-9

[7] Yaffe MB, Rittinger K, Volinia S, Caron PR, Aitken A et al. (1997) The structural basis for 14-3-3:phosphopeptide binding specificity. Cell 91: 961-971. doi:10.1016/S0092-8674(00)80487-0. PubMed: 9428519. 9428519 10.1016/s0092-8674(00)80487-0

[8] Liu D, Bienkowska J, Petosa C, Collier RJ, Fu H et al. (1995) Crystal structure of the zeta isoform of the 14-3-3 protein. Nature 376: 191-194. doi:10.1038/376191a0. PubMed: 7603574. 7603574 10.1038/376191a0

[9] Boston PF, Jackson P, Thompson RJ (1982) Human 14-3-3 protein: radioimmunoassay, tissue distribution, and cerebrospinal fluid levels in patients with neurological disorders. J Neurochem 38: 1475-1482. doi:10.1111/j.1471-4159.1982.tb07928.x. PubMed: 7062063. 7062063 10.1111/j.1471-4159.1982.tb07928.x

[10] Ge F, Li WL, Bi LJ, Tao SC, Zhang ZP et al. (2010) Identification of novel 14-3-3zeta interacting proteins by quantitative immunoprecipitation combined with knockdown (QUICK). J Proteome Res 9: 5848-5858. doi:10.1021/pr100616g. PubMed: 20879785. 20879785 10.1021/pr100616g

[11] Pozuelo Rubio M, Geraghty KM, Wong BH, Wood NT, Campbell DG et al. (2004) 14-3-3-affinity purification of over 200 human phosphoproteins reveals new links to regulation of cellular metabolism, proliferation and trafficking. Biochem J 379: 395-408. doi:10.1042/BJ20031797. PubMed: 14744259. 14744259 10.1042/BJ20031797 PMC1224091

[12] Bugg NC, Jones JA (1998) Hypophosphataemia. Pathophysiology, effects and management on the intensive care unit. J Anesth 53: 895-902. doi:10.1046/j.1365-2044.1998.00463.x. 10.1046/j.1365-2044.1998.00463.x 9849285

[13] Burt CT, Glonek T, Bárány M (1977) Analysis of living tissue by phosphorus-31 magnetic resonance. Science 195: 145-149. doi:10.1126/science.188132. PubMed: 188132. 188132 10.1126/science.188132

[14] Koretsune Y, Corretti MC, Kusuoka H, Marban E (1991) Mechanism of early ischemic contractile failure. Inexcitability, metabolite accumulation, or vascular collapse? Circ Res 68: 255-262. doi:10.1161/01.RES.68.1.255. PubMed: 1984866. 1984866 10.1161/01.res.68.1.255

[15] Dawson MJ, Gadian DG, Wilkie DR (1980) Mechanical relaxation rate and metabolism studied in fatiguing muscle by phosphorus nuclear magnetic resonance. J Physiol 299: 465-484. PubMed: 6966688. 6966688 10.1113/jphysiol.1980.sp013137 PMC1279237

[16] Burt CT, Glonek T, Bárány M (1976) Analysis of phosphate metabolites, the intracellular pH, and the state of adenosine triphosphate in intact muscle by phosphorus nuclear magnetic resonance. J Biol Chem 251: 2584-2591. PubMed: 4452. 4452

[17] Sluchanko NN, Artemova NV, Sudnitsyna MV, Safenkova IV, Antson AA et al. (2012) Monomeric 14-3-3ζ has a chaperone-like activity and is stabilized by phosphorylated HspB6. Biochemistry 51: 6127–6138. doi:10.1021/bi300674e. PubMed: 22794279. 22794279 10.1021/bi300674e PMC3413243

[18] Athwal GS, Huber JL, Huber SC (1998) Phosphorylated nitrate reductase and 14-3-3 proteins. Site of interaction, effects of ions, and evidence for an amp-binding site on 14-3-3 proteins. Plant Physiol 118: 1041-1048. doi:10.1104/pp.118.3.1041. PubMed: 9808749. 9808749 10.1104/pp.118.3.1041 PMC34777

[19] Pozdeyev N, Taylor C, Haque R, Chaurasia SS, Visser A et al. (2006) Photic regulation of arylalkylamine N-acetyltransferase binding to 14-3-3 proteins in retinal photoreceptor cells. J Neurosci 26: 9153-9161. doi:10.1523/JNEUROSCI.1384-06.2006. PubMed: 16957072. 16957072 10.1523/JNEUROSCI.1384-06.2006 PMC6674502

[20] Mymrikov EV, Seit-Nebi AS, Gusev NB (2011) Large potentials of small heat shock proteins. Physiol Rev 91: 1123-1159. doi:10.1152/physrev.00023.2010. PubMed: 22013208. 22013208 10.1152/physrev.00023.2010

[21] Sluchanko NN, Sudnitsyna MV, Seit-Nebi AS, Antson AA, Gusev NB (2011) Properties of the monomeric form of human 14-3-3zeta protein and its interaction with tau and HspB6. Biochemistry 50: 9797-9808. doi:10.1021/bi201374s. PubMed: 21978388. 21978388 10.1021/bi201374s

[22] Sluchanko NN, Sudnitsyna MV, Chernik IS, Seit-Nebi AS, Gusev NB (2011) Phosphomimicking mutations of human 14-3-3zeta affect its interaction with tau protein and small heat shock protein HspB6. Arch Biochem Biophys 506: 24-34. doi:10.1016/j.abb.2010.11.003. PubMed: 21081103. 21081103 10.1016/j.abb.2010.11.003

[23] Chernik IS, Seit-Nebi AS, Marston SB, Gusev NB (2007) Small heat shock protein Hsp20 (HspB6) as a partner of 14-3-3gamma. Mol Cell Biochem 295: 9-17. doi:10.1007/s11010-006-9266-8. PubMed: 17109079. 17109079 10.1007/s11010-006-9266-8

[24] Sluchanko NN, Seit-Nebi AS, Gusev NB (2009) Effect of phosphorylation on interaction of human tau protein with 14-3-3zeta. Biochem Biophys Res Commun 379: 990-994. doi:10.1016/j.bbrc.2008.12.164. PubMed: 19138662. 19138662 10.1016/j.bbrc.2008.12.164

[25] Brown PH, Schuck P (2006) Macromolecular size-and-shape distributions by sedimentation velocity analytical ultracentrifugation. Biophys J 90: 4651-4661. doi:10.1529/biophysj.106.081372. PubMed: 16565040. 16565040 10.1529/biophysj.106.081372 PMC1471869

[26] Laue TM, Shah BD, Ridgeway TM, Pelletier SL (1992) Analytical Ultracentrifugation in Biochemistry and Polymer Science. Cambridge: Royal Society of Chemistry. 90pp.

[27] Ge Q, Huang N, Wynn RM, Li Y, Du X et al. (2012) Structural characterization of a unique interface between carbohydrate response element-binding protein (ChREBP) and 14-3-3beta protein. J Biol Chem 287: 41914-41921. doi:10.1074/jbc.M112.418855. PubMed: 23086940. 23086940 10.1074/jbc.M112.418855 PMC3516738

[28] Bustad HJ, Skjaerven L, Ying M, Halskau O, Baumann A et al. (2012) The peripheral binding of 14-3-3gamma to membranes involves isoform-specific histidine residues. PLOS ONE 7: e49671. doi:10.1371/journal.pone.0049671. PubMed: 23189152. 23189152 10.1371/journal.pone.0049671 PMC3506662

[29] Bustos DM, Iglesias AA (2005) A model for the interaction between plant GAPN and 14-3-3zeta using protein–protein docking calculations, electrostatic potentials and kinetics. J Mol Graph Modell 23: 490-502. doi:10.1016/j.jmgm.2005.03.002. 10.1016/j.jmgm.2005.03.002 15896993

[30] Camoni L, Di Lucente C, Pallucca R, Visconti S, Aducci P (2012) Binding of phosphatidic acid to 14-3-3 proteins hampers their ability to activate the plant plasma membrane H+-ATPase. IUBMB Life 64: 710-716. doi:10.1002/iub.1058. PubMed: 22715055. 22715055 10.1002/iub.1058

